# The Association of Mean Platelet Volume with Intra Ventricular Hemorrhage and Broncho Pulmonary Dysplasia in Preterm Infants

**Published:** 2015-12-10

**Authors:** K Bolouki Moghaddam, M Zarkesh, A Kamali, S Dalili, A Heidarzadeh, A Hassanzadeh Rad

**Affiliations:** 1 Pediatric growth disorders research center, 17 shahrivar hospital, school of medicine, Guilan University of medical sciences, Rasht, Iran; 2 School of Medicine, Guilan University of Medical Sciences, Iran

**Keywords:** Bronchopulmonary dysplasia, Intraventricular hemorrhage, Mean platelet volume, preterm infants

## Abstract

**Background:**

Interventricular hemorrhage (IVH) and Broncho pulmonary dysplasia(BPD) commonly occur in premature infants and they associate with platelet dysfunction. The aim of this study was to investigate the association of MPV and occurrence of IVH and BPD.

**Materials and methods:**

In this cross sectional study, 3 groups including IVH, BPD and control were compared. All participants were preterm neonates with <35 weeks of gestation. MPV was recorded during the first 48 hours of life for all cases. Data were reported by descriptive statistics and analyzed by Pearson correlation coefficient, spearman correlation coefficient, paired T test and multinomial regression analysis in SPSS version 17.

**Results:**

Higher MPV level was noted in BPD and IVH groups versus control group (9.79±0.73 fl and 10±1.04 fl versus 8.33±0,91 fl p<0.0001). Also, most participants in BPD (93.3%) and IVH (73.3%) groups had MPV >9 fl compared to controls (16.7%) (p<0.0001). Regression analysis showed that only MPV related to the occurrence of IVH (OR=2.200 95%CI p=0.013) and elevated MPV significantly increased duration of O2 therapy (p<0.0001) and mechanical ventilation (p=0.0010).

**Conclusion:**

MPV value at first 48 hours of life can be noted as a simple biomarker for occurrence of BPD and specially IVH in preterm infants

## Introduction

Assessing platelets in the neonatal period can indicate several functions both in physiological and pathological conditions including; homeostasis, integrity of blood vessels, transportation and phagocytosis.(1) The mean platelet volume (MPV) averages from 7 to 9 fl for both full- term and preterm neonates.(2, 3) Complete blood count easily provides platelet volume data (4). In adults, MPV level may be elevated in inflammation such as acute myocardial infarction (5). In premature infants, Bronchopulmonary dysplasia (BPD) and intraventricular hemorrhage (IVH) are two neonatal morbidities. Their risks are inversely related to both gestational age and weight at birth. Previous studies mentioned possible correlation between MPV and occurrence of BPD(6, 7) and IVH in preterm infants.(4)Although High MPV may favor inflammatory and oxidative lung damages(6), coagulation and platelet function have significant roles in the pathogenesis of IVH (8) and larger platelets are more reactive and may be associated with shortened bleeding times,(9) yet it is a controversial issue.(7)The aim of this study was to investigate the association of MPV and occurrence of IVH and BPD.

## Materials and Methods

This is a cross sectional study with control group carried out at neonatal ward and neonatal intensive care unit of 17 shahrivar children hospital Rasht – Iran from April 2011 to April 2015. The studied group consisted of 30 infants with BPD, 30 infants with IVH, and 30 infants without IVH and BPD (control group). All participants were preterm neonates with <35 weeks of gestation. According to the large number of patients with IVH and control groups, an anonymous clinician randomly selected these patients during the study period. Infants with thrombocytopenia (platelet<150,000 ×109/L), congenital malformations and/or metabolic disorders were excluded. Data were gathered by a form including sex, gestational age, type of delivery, birth weight, surfactant and mechanical ventilation need, duration of oxygen therapy and mechanical ventilation, and the MPV level. Blood samples were drawn from each infant by arterial puncture during the first 48 hours of life. Platelet count and MPV determination were performed on the Sysmex coulter counter model (Japan). 

BPD defined as the need for supplemental oxygen at postnatal 28th day of life (10) and IVH defined by cranial ultrasound findings. 

**Statistical analysis**

Data were reported by descriptive statistics (mean and standard deviation) and analyzed by pearson correlation coefficient, spearman correlation, paired T test and multinomial regression analysis test in SPSS version 17. P value <0.05 was considered statistically significant and 95% confidence interval was noted.

## Results

Demographic, clinical and laboratory results of groups are summarized in table 1. Results showed significant difference between BPD and control group regarding gestational age, birth weight, mechanical ventilation and surfactant need, duration of oxygen therapy and MPV(p<0.05). Also, there were significant difference between IVH and control groups regarding gestational age, birth weight, type of delivery, surfactant need, and duration of oxygen therapy.(p<0.05) MPV level was higher in BPD and IVH groups versus control group(P<0.0001). Multivariate stepwise logistic regression analysis of selected variables in BPD and IVH groups demonstrated that only MPV related to the occurrence of IVH (OR=2.200 95%CI P=0.013).

**Figure 1:**
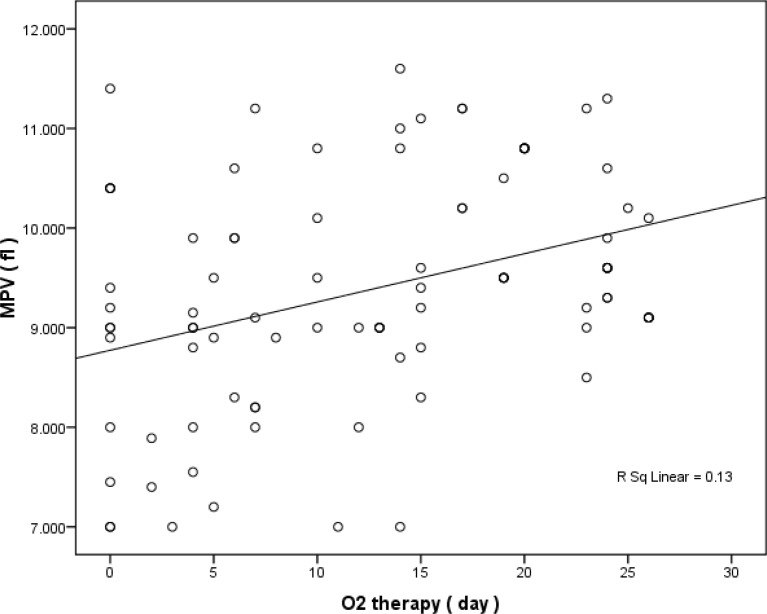
Relationship between MPV and Duration of o2 therapy( day

**Figure 2:**
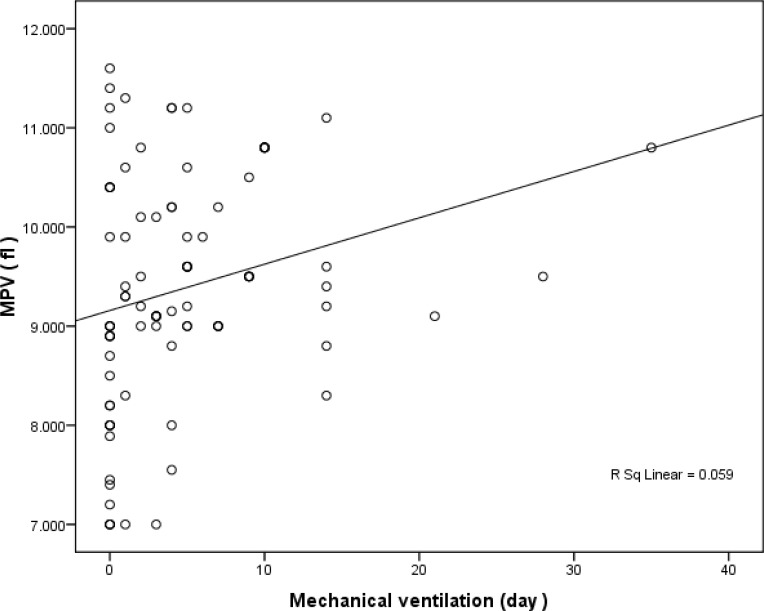
Relationship between MPV and Duration of Mechanical ventilation ( day

Furthermore, elevated MPV level significantly increased duration of O2 therapy ( pearson correlation =361 pvalue < 0.0001) and mechanical ventilation ( spearman correlation =0.334 Pvalue =0.001) (Figures 1 and 2).

**Table I:** Distribution of nosocomial infections among nosocomial positive patients

		**IVH (N=30)**	**BPD (N=30) **	**non BPD- non IVH (control** **) (N=30)**	**P value**
**Gestational age (week)**	**Mean** **±** **SD**	**2.43** **±** **29.60**	**0.997** **±** **29.80**	**1.88** **±** **32.40**	**<0.0001** 1
					**<0.0001** 2
					**<0.0001** 3
**Birth ** **Weight(gr)**	**Mean** **±** **SD**	**313.14** **±** **1306/67**	**139.99** **±** **1194**	**1797 ±401.02**	**<0.0001** 1
					**<0.0001** 2
					**<0.0001** 3
**Sex : ** ** male(N)** **Female**	**N(%)**	**10(33.3%)** **20(66/7%)**	**12(40%)** **18(60%)**	**17(56.6%)** **13(43.3%)**	**0.069** 1
					**0.196** 2
					**0.171** 3
**Mode of delivery:** **NVD C/S**	**N(%)**	**-** **30(100%)**	**6(20%)** **24(80%)**	**6(20%)** **24(80%)**	**0.024** 1
					**1.0** 2
					**0.02** 3
**Need to MV(N)** **No** **Yes**	**N(%)**	**14(46.7%)** **16(53.4%)**	**30(100%)** ** -**	**10(33.3%)** **20(66.7%)**	**0.29** 1 **<0.0001** 2 **<0.0001** 3
**need for surfactants(N)** **No** **Yes**	**N(%)**	**14(46.6%)** **16(53.4%)**	**12(40%)** **18(60%)**	**22(73.3%)** **8(26.7%)**	**0.035** 1 **0.009** 2 **0.024** 3
**O**_2_ ** therapy(day)**	**Mean** **±** **SD**	**15/16±9.5 1**	**29.86±3.73**	**6.23±5.51**	**<0.0001** 1 **<0.0001** 2 **<0.0001** 3
**MPV(fl)**	**Mean** **±** **SD**	**1.04** **±** **10.00**	**0.73** **±** **9.79**	**0.91** **±** **8.33**	**<0.0001** 1 **<0.0001** 2 **<0.0001** 3
**MPV** **≤9fl** **>9 fl**	**N(%)**	**8(26/7%)** **22(73/3%)**	**2(6.7%)** **28(93.3%)**	**25(83/3%)** **5(16.7%)**	**<0.0001** 1 **<0.0001** 2 **<0.0001** 3

MV=Mechanical ventilation

MPV=Mean platelet volume

1 =P value between IVH and control group

2 = P value between BPD and control group

3 =Overall P value

## Discussion

Some recent studies suggested that MPV might predict the development of respiratory distress syndrome(RDS), BPD, IVH, necrotizing entrocolitis, and sepsis in preterm infants. (4, 11-13) We found that MPV valves were significantly higher in IVH and BPD groups than control one. Our results showed that preterm infants with BPD significantly need more O2therapy and mechanical ventilation as compared to control group. These findings were in accordance with Hussein et al in Egypt (13) and Dani et al in Italy (7). They reported significantly higher need to mechanical ventilation and O2 therapy in preterm infants with BPD (P<0/001) .

In the current study, MPV>9fl was significantly more in BPD group compared to the control group (P<0.0001 ). Dani et al and Hussein et al reported that MPV>11 fl was significantly higher in BPD than non-BPD group (7, 13). Dani et al noted that higher MPV might favor development of BPD by worsening respiratory distress syndrome through surfactant inhibition (7). Disruption of alveolocapillary membrane integrity in RDS resulted in leakage of coagulation factors into alveolar spaces. High levels of activated procoagulant factors and insufficient fibrinolysis leaded alveolar fibrin deposition and hyaline membrane formation in infants with higher MPV(14). Fibrinogen and related products are known to be potent inhibitors of surfactant(15). Cekmez et al in Turkey found that higher MPV in the first hours of life might be a risk factor for occurrence of BPD and IVH in preterm neonates with <34 weeks of gestation. They mentioned that higher MPV may be associated with inflammatory and oxidative processes. They noted neonatal diseases such as BPD and IVH as multi factorial problems(4).

 In the light of our study, MPV at first 48 hours of life was significantly higher in preterm infants with IVH than control group. This was inconsistent with Dani et al who assessed preterm infants with <30 weeks of gestation. They found similar MPV level in premature infants with and without IVH (P= 0.256) (7). Hussein et al reported that MPV at 24-48 hours of life was significantly higher in patients with IVH versus non-IVH group (p<0.001) (13).

 In our study by logistic regression test only MPV was noted as an independent risk factor for development of IVH. Also Hussein et al which assessed very low birth weight infants mentioned the same results. They noted MPV>11 fl as an independent risk factor which increased the risk of IVH (not BPD) with OR=65.6 95%CI (p<0.001). In addition, they noted male gender, birth weight<1250gr and Apgar score<8 at 5th minute as other independent risk factors of IVH (13). But Nouripoor et al indicated higher MPV during the first 72 hours of life as an independent risk factor for occurrence of BPD in preterm infants with RDS (OR=1.6 , 95% CI:1.08-2.38 p=0.019). They didn't assess the relationship between MPV and IVH(6).

Increased MPV elevated duration of o2 therapy and mechanical ventilation significantly. To our knowledge, this is the first study which investigated the possible relationship between MPV and duration of o2 therapy and mechanical ventilation.

According to our results, it seems that preterm infants with higher MPV in the first 48 hours of life need better care to prevent IVH and BPD. Therefore, assessing risk factors of IVH and BPD such as monitoring blood pressure and ventilator setting can be recommended.

The limitation of this study was the inability to evaluate MPV with BPD stages and IVH grades. We also could not follow up further MPV changes after 48 h of birth.

## Conclusion

MPV value at first 48 hours of life can be noted as a simple biomarker for occurrence of BPD and specially IVH in preterm infants 
